# Big Genes, Small Effectors: Pea Aphid Cassette Effector Families Composed From Miniature Exons

**DOI:** 10.3389/fpls.2020.01230

**Published:** 2020-09-02

**Authors:** Matthew Dommel, Jonghee Oh, Jose Carlos Huguet-Tapia, Endrick Guy, Hélène Boulain, Akiko Sugio, Marimuthu Murugan, Fabrice Legeai, Michelle Heck, C. Michael Smith, Frank F. White

**Affiliations:** ^1^ Department of Plant Pathology, University of Florida, Gainesville, FL, United States; ^2^ Department of Plant Pathology, Kansas State University, Manhattan, KS, United States; ^3^ INRAE, UMR Institute of Genetics, Environment and Plant Protection, Le Rheu, France; ^4^ Department of Entomology, Kansas State University, Manhattan, KS, United States; ^5^ USDA-ARS, Cornell University, Ithaca, NY, United States

**Keywords:** pea aphid, *Acyrthosiphon pisum*, salivary gland, secretion protein, effector protein, gene family, proteomic analysis

## Abstract

Aphids secrete proteins from their stylets that evidence indicates function similar to pathogen effectors for virulence. Here, we describe two small candidate effector gene families of the pea aphid, *Acyrthosiphon pisum*, that share highly conserved secretory signal peptide coding regions and divergent non-secretory coding sequences derived from miniature exons. The KQY candidate effector family contains eleven members with additional isoforms, generated by alternative splicing. Pairwise comparisons indicate possible four unique KQY families based on coding regions without the secretory signal region. KQY1a, a representative of the family, is encoded by a 968 bp mRNA and a gene that spans 45.7 kbp of the genome. The locus consists of 37 exons, 33 of which are 15 bp or smaller. Additional KQY members, as well as members of the KHI family, share similar features. Differential expression analyses indicate that the genes are expressed preferentially in salivary glands. Proteomic analysis on salivary glands and saliva revealed 11 KQY members in salivary proteins, and KQY1a was detected in an artificial diet solution after aphid feeding. A single KQY locus and two KHI loci were identified in *Myzus persicae*, the peach aphid. Of the genes that can be anchored to chromosomes, loci are mostly scattered throughout the genome, except a two-gene region (KQY4/KQY6). We propose that the KQY family expanded in *A. pisum* through combinatorial assemblies of a common secretory signal cassette and novel coding regions, followed by classical gene duplication and divergence.

## Introduction

Aphids are important pests of plants that can cause economical damage through loss of crop yield and dissemination of plant viruses through their feeding habits (Miles, 1999). There are many different species of aphid that have been found to cause crop damage, including the pea aphid, *Acyrthosiphon pisum*. The various species of aphid all display a diversity of host ranges, extending from narrow to broad (Jaouannet et al., 2014). An aphid with a narrow host range consumes either one individual species or closely related plants within a single family. An aphid with a broad host range can feed on many different plant species spanning different taxonomic families. During this interaction, aphids extract phloem sap from the leaves and stems of the host plant through stylets, which are inserted into phloem cells. Plants possess both a constitutive and inducible immune response that fights insect consumption (Cook et al., 2015). Once fed upon, a plant can mount a defensive response to thwart parasitic processes. Aphid interactions with non-host plants are hypothesized to fail, in part, due to an immune reaction, while a successful aphid feeding involves suppressing the plant immune response (Jaouannet et al., 2014).

During feeding, aphids secrete saliva, which contains numerous proteins, enzymes, and other compounds, that assist stylet insertion, nutrient extraction, and host tissue interactions (Miles, 1999; Tjallingii, 2006; Will et al., 2007). Upon probing of a potential feeding plant, aphids secrete gelling saliva that acts to surround and protect the stylet. After puncturing the plant, the aphids secrete a watery saliva to thwart plant defenses (Miles, 1999). Components of the salivary proteins are hypothesized to play a role in facilitating the interaction with the host, in analogy to effectors of plant pathogenic bacteria and fungi. In contrast to pathogen effectors, functional evidence for effector action is limited in aphids. Nonetheless, variations in candidate effectors of aphids are hypothesized to contribute to the adaptation of aphid populations to specific host species (biotypes) and genotypes. Ectopic expression and silencing of some candidate aphid effectors have been shown to affect aphid fecundity and growth on host plants (Mutti et al., 2008; Pitino and Hogenhout, 2013).

Effector proteins are often relatively small proteins with no clear function based on relatedness to other proteins and are secreted into the host cell or extracellular milieu. A prominent example of a pea aphid effector is the protein C002. Identified initially from an EST library from the salivary glands, C002 is secreted into the target plant and hypothesized to assist in feeding (Mutti et al., 2008). Reduced expression through inhibitory RNA (RNAi) of the C002 transcript resulted in reduced feeding time of the aphids and, ultimately, premature death. Since the discovery of C002, C002 homologs and additional candidate aphid effectors have been identified (Elzinga and Jander, 2013; Rodriguez and Bos, 2013; Chaudhary et al., 2015; Thorpe et al., 2016; Boulain et al., 2018). One effector of *M. persicae*, Mp10, has been immunologically localized to the cytoplasm and chloroplasts of plant cells (Mugford et al., 2016).

The pea aphid is a model aphid species that exhibits a narrow host range feeding on legumes exclusively. The pea aphid genome and multiple other aphid genomes are available for analysis and comparisons (Richards et al., 2010; Burger and Botha, 2017; Wenger et al., 2017; Chen et al., 2019; Li et al., 2019; Quan et al., 2019). Additional genomic resources and salivary gland expressed sequence tag (EST) libraries of *A. pisum*, and other phytophagous aphids, provide numerous effector candidates (International Aphid Genomics Consortium, 2010; Legeai et al., 2010; Shigenobu et al., 2010). Additionally, mass spectrometry proteomic analysis has been used to identify these proteins from salivary glands tissue and saliva secreted into artificial diets (Carolan et al., 2009; Cooper et al., 2010; Carolan et al., 2011; Rao et al., 2013; Chaudhary et al., 2015; Boulain et al., 2018). Despite this progress, much remains to be known about the effectors of aphid salivary proteins in aphid-host plant interactions (Mutti et al., 2006; Carolan et al., 2011; Rao et al., 2013; Boulain et al., 2018). Here, we report the identification of two candidate effector gene families of *A. pisum* and *M. persicae*.

## Results

### Identification of Cassette Gene Families in Pea Aphid

**Figure 1:**
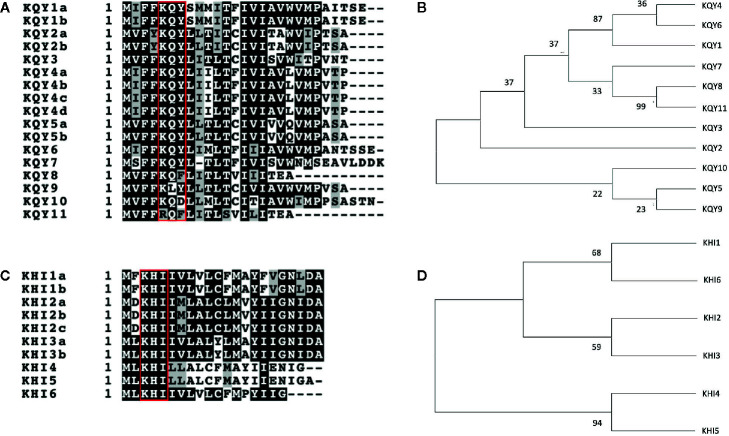
Alignment and phylogeny of KQY and KHI members. **(A, B)** Amino acid sequence alignment of the N-terminal regions of KQY **(A)** and KHI **(B)** gene families, respectively. The alignments were produced with the ClustalW multiple alignment program. The reverse-shaded amino acids represent identical amino acid residues among member of the gene family. The red squares highlight the conserved residues from which the names KQY and KHI were derived. **(C, D)** Phylogeny based on the nucleic acid sequence of the signal peptides of the KQY **(C)** and KHI **(D)** gene families. Maximum-likelihood tree with numbers next to the branches showing bootstrap values as a percentage out of 1,000 replicates.

Previously sequenced salivary gland cDNA sequences for *A. pisum* were retrieved from NCBI, and dataset was analyzed for sequences encoding predicted secreted peptides. Multiple transcripts were identified that encoded relatively short (100–450 aa) proteins and, upon alignment, could be divided into two families based on predicted amino acid sequence similarities (**Supplemental Figures 1** and **2**). Each family, named KQY and KHI, was composed of multiple genes and, in some cases, two to four isoforms, which were produced by alternative splicing (**Table 1**). At least one member of the loci, with the exception of KQY2, were found previously to be up-regulated in salivary glands (**Table 1**, Boulain et al., 2018). Three related sequences were also identified in the peach aphid (*Myzus persicae*) genome (**Table 1**). The notable feature of the predicted proteins is the conserved signal peptide region, ranging in size from 19 to 28 amino acids, combined with C-terminal divergent sequences (**Figures 1A, B**). The families were, hereafter, referred to as candidate cassette effectors, and the two families were named KQY and KHI after conserved amino acid sequences in the N-terminal region of all or most members (**Figures 1A, B**). The KQY family is comprised of eleven genes and seventeen different isoforms due to splicing variants (**Table 1**). One member was identified in *M. persicae*. The KHI family is composed of six genes and 10 isoforms. Two members were identified in *M. persicae*. The proteins range from 9.2 to 24.4 kDa.

**Table 1:** Members of the KQY and KHI families.

Gene Family	Gene/Isoform^a^	Gene Locus	Transcript ID	SG Up^b^	Protein ID	Signal Peptide Predication(TargetP-2.0)^c^
**KQY**	KQY1a	LOC100158789 ACYPI000223	NM_001162442	+	NP_001155914	MIFF**KQY**SMMITFIVIAVWVMPAITSE (0.8578)
	KQY1b	LOC100158789 ACYPI000223	AK339882		BAH70584	MIFF**KQY**SMMITFIVIAVWVMPAITSE (0.855)
	KQY2a	LOC100302371	AK342599		BAH72568	MVFY**KQY**LLTITCIVITAWVIPTSA (0.9902)
	KQY2b	LOC100302371	AK342927		BAH72760 NP_001156348	MVFY**KQY**LLTITCIVITAWVIPTSA (0.9902)
	KQY3	LOC100301916 ACYPI073633	AK341661	+	BAH72003 NP_001153885	MVFF**KQY**LITLTCIVISVWITPVNT (0.9924)
	KQY4a	LOC100302370	AK342948	+	BAH72770 NP_001156343	MIFF**KQY**LIILTFIVIAVLVMPVTP (0.9362)
	KQY4b	LOC100302370	AK342242		BAH72349	MIFF**KQY**LIILTFIVIAVLVMPVTP (0.9324)
	KQY4c	LOC100302370	AK342690		BAH72627	MIFF**KQY**LIILTFIVIAVLVMPVTP (0.9297)
	KQY4d	LOC100302370	AK342678		BAH72619	MIFF**KQY**LIILTFIVIAVLVMPVTP (0.9537)
	KQY5a	LOC100302375	AK342406	+	BAH72443 NP_001156434	MVFF**KQY**LLTLTCIVIVVQVMPASA (0.9963)
	KQY5b	LOC100302376	AK342378		BAH72425 NP_001156435	MVFF**KQY**LLTLTCIVIVVQVMPASA (0.9857)
	KQY6	LOC100302481 (NV12)	AK340126	+	BAH70788 NP_001156817	MIFF**KQY**LIMLTFIIIAVWVMPANT (.9693)
	KQY7	LOC100302485 (NV22)	AK340563	+	BAH71149 NP_001156835	MSFF**KQY**LTLTFIVISVWNMSEA (0.9649)
	KQY8	LOC100302439 ACYPI24906	AK342473	+	BAH72486 NP_001156591	MVFF**KQF**LITLTVIIITEA (0.9676)
	KQY9	LOC100302403	AK342683	+	BAH72621 NP_001156509	MVFF**KLY**LLTLTCIVIAVWVMPVSA (0.9981)
	KQY10	LOC100302381	AK342808	+	BAH72693 NP_001156441	MFNVLIILSLISYTFEPSYTLYKFKMVFF**KQD**LLMLTCITIAVWIMPPSASTN (0.8081)
	KQY11	LOC100302480	AK340121	+	BAH70784 NP_001156816	MVFF**RQF**LITLSVILITEA (0.9787)
	KQY^Mp^	LOC111039170 (*Myzus persicae*)	XM_022322515		XP_022178207	MHFF**KHY**LIVLTYIVISFWFMPSASL (0.9345)
**KHI**	KHI1a	LOC100159750 ACYPI001099	AK339863	+	BAH70570 NP_001155863	MF**KHI**IVLVLCFMAYFVGNLDA (0.998)
	KHI1b	LOC100159750 ACYPI001099	AK339862		BAH70569	MF**KHI**IVLVLCFMAYFVGNLDA (0.9983)
	KHI2a	LOC100302383	AK341162	+	BAH71618	MD**KHI**IMLALCLMVYIIGNIDA (0.9936)
	KHI2b	LOC100302383	AK341161		BAH71617 NP_001156448	MD**KHI**IMLALCLMVYIIGNIDA (0.9937)
	KHI2c	LOC100302383	AK342769		BAH72672	MD**KHI**IMLALCLMVYIIGNIDA (0.9952)
	KHI3a	LOC100166702 ACYPI007553	AK340197	+	BAH70850 NP_001156548	ML**KHI**IVLALYLMAYIIGNIDA (0.9965)
	KHI3b	LOC100166702 ACYPI007553	AK342603		BAH72572 NP_001155718	ML**KHI**IVLALYLMAYIIGNIDA (0.9947)
	KHI4	LOC100570519 ACYPI46154	AK341077	+	BAH71554 NP_001280397	ML**KHI**LLALCFMAYIIENIG (0.9586)
	KHI5	LOC100534636	AK341390	+	BAH71796 NP_001191953	ML**KHI**LLALCFMAYIIENIGA (0.9976)
	KHI6	LOC100571631	AK340760	+	BAH71306 NP_001233103	ML**KHI**IVLVLCFMPYIIG (0.9985)
	KHI^Mp^1a	LOC111029516	XM_022308527	nd	XP_022164219	MV**RHI**IMLAICIMFYIIGNAMALTPAERKA
	KHI^Mp^1b	LOC111029516	XM_022308528	nd	XP_022164220	MV**RHI**IMLAICIMFYIIGNAMALTPAERKA
	KHI^Mp^1c	LOC111029516	XM_022308529	nd	XP_022164221	MV**RHI**IMLAICIMFYIIGNAMALTPAERKA
	KHI^Mp^2a	LOC111029518	XM_022308532	nd	XP_022164224	MSTMV**KHI**NMLALFIMFYIIGNAMALTPAERKA
	KHI^Mp^2b	LOC111029518	XM_022308533	nd	XP_022164225	MSTMV**KHI**NMLALFIMFYIIGNAMALTPAERKA
	KHI^Mp^2c	LOC111029518	XM_022308534	nd	XP_022164226	MSTMV**KHI**NMLALFIMFYIIGNAMALTPAERKA
	KHI^Mp^2d	LOC111029518	XM_022308536	nd	XP_022164228	MSTMV**KHI**NMLALFIMFYIIGNAMALTPAERKA
**C002**	C002^Ap^	LOC100167863 ACYPI008617	XM_001948323	+	XP_001948358	MGSYKLYVAVMAIAIAVVQEVRC (0.9704)

a Mp, Myzus persicae, Ap, Acyrthosiphon pisum.

b +, Identified as up-regulated in salivary glands in comparison to alimentary tract by Boulain et al. (2018). Up-regulation of locus is indicated, and no differential expression of isoforms is implied. nd, not detected, no salivary gland ESTs from M. persicae were identified by BLAST.

c KQY10 and KHI^Mp^ isoforms predicted N-terminal peptide, which may be misannotated.

A maximum likelihood phylogeny was produced, using the N-terminal nucleotides coding sequences for signal peptide region that is unique for each gene (**Figures 1C, D**). Two distinct groups of KQY genes cluster together through high bootstrap values; KQY1, KQY4, KQY6, and KQY8, KQY11. KQY1, KQY4, and KQY6 possess a related bootstrap value of 87, though the KQY4 and KQY6 are more distantly related within this group, only containing a bootstrap value of 36. KQY8 and KQY11 are highly similar, which is related in their bootstrap value of 99. Beyond the secretory signal peptide coding region, pairwise BLAST analysis of the KQY coding sequences indicates four possible gene families (KQY1, 4, 6; KQY2, 5, 9, 10; KQY3, 8, 11, Mp; KQY 7) at the probability level of 1 x 10^-5^(**Supplemental Figure 3**). KQY7 shares no sequence relatedness beyond the secretory signal region at the DNA or protein level. At the same time, all members share some sequence identity in 3’ region of the transcripts at the nucleotide level, with the exception of KQY7 and KQYMp (**Supplemental Figure 4**).

Similarly, within the KHI gene family, only two KHI members cluster close together according to bootstrap values, KHI4 and KHI5. KHI4 and KHI5 possess a bootstrap value of 94, indicated high homology. The remaining the KHI gene signal peptides are loosely related with KHI1, KHI6, and KHI2, KHI3 clustering with bootstrap values of 68 and 59, respectively.

KQY10 is annotated with twenty-nine additional N-terminal amino acid residues in comparison to the other family members (**Table 1**). Both the sequence, as annotated, or a shortened version are predicted to contain a signal peptide. Similarly, the KHI^Mp^2 locus, including all isoforms, are annotated with three additional amino acid residues at the N-terminus (**Table 1**).

### Identification of Candidate Cassette Effectors by Proteomic Analysis of Salivary Gland Proteins

**Figure 2:**
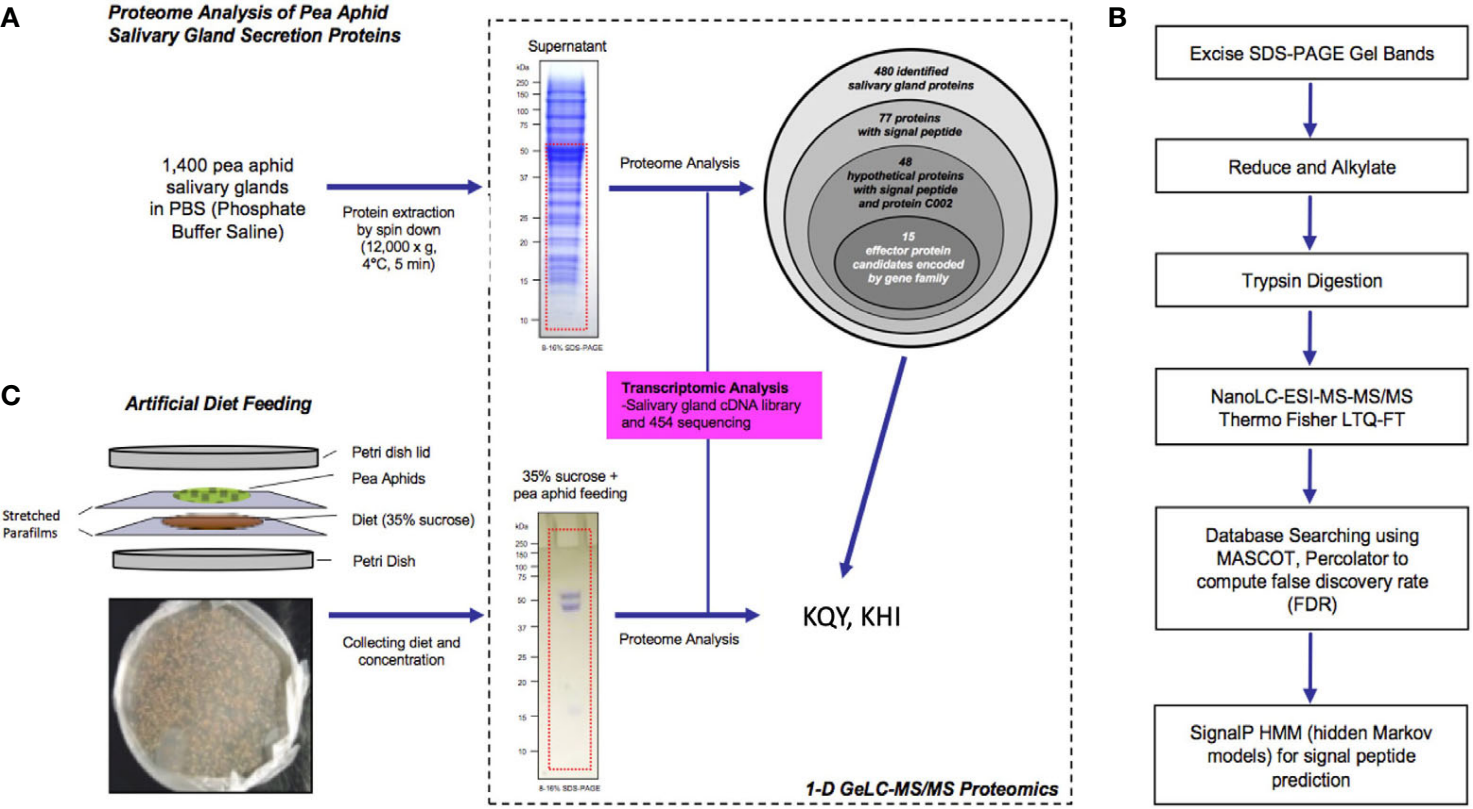
Proteomic analysis of pea aphid salivary gland secretion proteins. **(A)** Schematic representation of secretion proteome analysis of pea aphid salivary gland. **(B)** 1-D GeLC-MS/MS Proteomics flowchart to identify salivary gland secretion proteins. **(C)** Schematic of artificial diet feeding experiment.

A proteomic analysis was conducted to determine whether member of the two families were present in salivary glands and secreted in salivary fluids (**Figure 2A**). Proteins were extracted from the salivary gland tissues of *A. pisum* and separated on a SDS-PAGE (1DE) gel (**Figure 2B**). Proteins in 10–60 kDa range were then subjected to 1-D GeLC-MS/MS. Of the 480 proteins, 77 proteins with predicted secretion signals were present (**Supplementary Table 1**). Notably, 16 of the candidate secreted gland proteins were members of the KQY and KHI families (**Table 2**; **Supplemental Table 1**). Ten KQY proteins were found, namely KQY1a, KQY2a, KQY2b, KQY3, KQY4a, KQY4c, KQY5b, KQY9, KQY10, and KQY11. KQY2a and b, KQY4a and c, and KHI2a and b were the isoforms found concurrently. Five of the KHI gene family corresponding proteins of the six KHI genes were identified, and six out of the ten protein isoforms were found (**Table 2**, **Supplemental Table 1**). In addition to other candidate effectors, the analysis identified the conserved aphid effector C002 (**Table 2**).

**Table 2:** Cassette effectors from proteomic analysis *A. pisum* salivary glands.

Gene Family	Protein Name	NCBI Accession Number	# of Peptides (Unique)	% Coverage
**KQY**	KQY1a ACYPI000223 LOC100158789	NP_001155914	17(14)	66
	KQY2a LOC100302371	BAH72568	9(8)	47
	KQY2b LOC100302371	NP_001156348	8(6)	36
	KQY3 LOC100301916	NP_001153885	2(1)	13
	KQY4a LOC100302370	NP_001156343	10(9)	43
	KQY4c LOC100302370	BAH72627	10(9)	45
	KQY5b LOC100302376	NP_001156435	8(8)	45
	KQY9 LOC100302403	NP_001156509	3(3)	32
	KQY9 LOC100302403	NP_001156509	3(3)	24
	KQY10 LOC100302381	NP_001156441	1(1)	6
	KQY11 LOC100302480	NP_001156816	3(3)	24
**KHI**	KHI1a ACYPI001099 LOC100159750	NP_001155863	4(3)	33
	KHI2a LOC100302383	BAH71618	2(2)	14
	KHI2b LOC100302383	NP_001156448	2(2)	14
	KHI3a LOC100166702	NP_001156548	12(9)	56
	KHI5 LOC100534636	NP_001191953	6(6)	38
	KHI6 LOC100571631	NP_001233103	4(4)	44
**C002**	C002	XM_001948323	5(4)	33

Proteins were collected from artificial diet media after feeding by *A. pisum* to determine if any of the family members could be detected in extracellular fluids using an artificial diet (**Figure 2C**). Total protein was analyzed through 1-D GeLC-MS/MS (**Figure 2B**). A total of nine aphid proteins were identified, including KQY protein, KQY1a (**Table 3**). Additional proteins included amino peptidase and angiotensin converting enzymes and have been previously observed (Boulain et al., 2018).

**Table 3:** *A. pisum* salivary gland proteins detected in synthetic diet using 1-D geLC-MS/MS.

Protein Name	NCBI Accession	Top Ion E-value	# of Peptides (Unique)	% Coverage	Signal Peptide Probability (D-score)	Mw (kDa)	Predicted Protein Name/Function
KQY1a	NP_001155863	2.10E-04	1(1)	8	0.645	23.1	KQY gene family
Aminopeptidase N-like	gi|193636568	2.20E-09	19(15)	51	0.822	70.1	M1 zinc dependent metalloprotease
Angiotensin converting enzyme-like	gi|193669489	5.20E-07	7(6)	42.9	0.703	74.2	M2 zinc dependent angiotensin converting enzyme (peptidase)
Angiotensin converting enzyme-like	gi|209571509	1.30E-04	4(4)	9.6	0.994	74.5	M2 zinc dependent angiotensin converting enzyme (peptidase)
Aminopeptidase N-like	gi|193702193	4.00E-06	18(9)	29.2	0.265	90	M1 zinc dependent metalloprotease-
Leucyl-cystinyl aminopeptidase-like	gi|193643503	3.30E-07	9(6)	31.5	0.974	105	M1 zinc dependent metalloprotease
Aminopeptidase N-like isoform 1	gi|193634323	9.00E-10	16(13)	59.8	0.991	105.2	M1 zinc dependent metalloprotease
Aminopeptidase N-like	gi|193657504	8.30E-06	5(3)	7.8	0.753	105.4	M1 zinc dependent metalloprotease
Aminopeptidase N-like	gi|193575561	2.80E-10	30(21)	31.6	0.981	105.5	M1 zinc dependent metalloprotease

### Large Gene Structure and Genomic Location of Candidate Cassette Effector Genes

**Figure 3:**
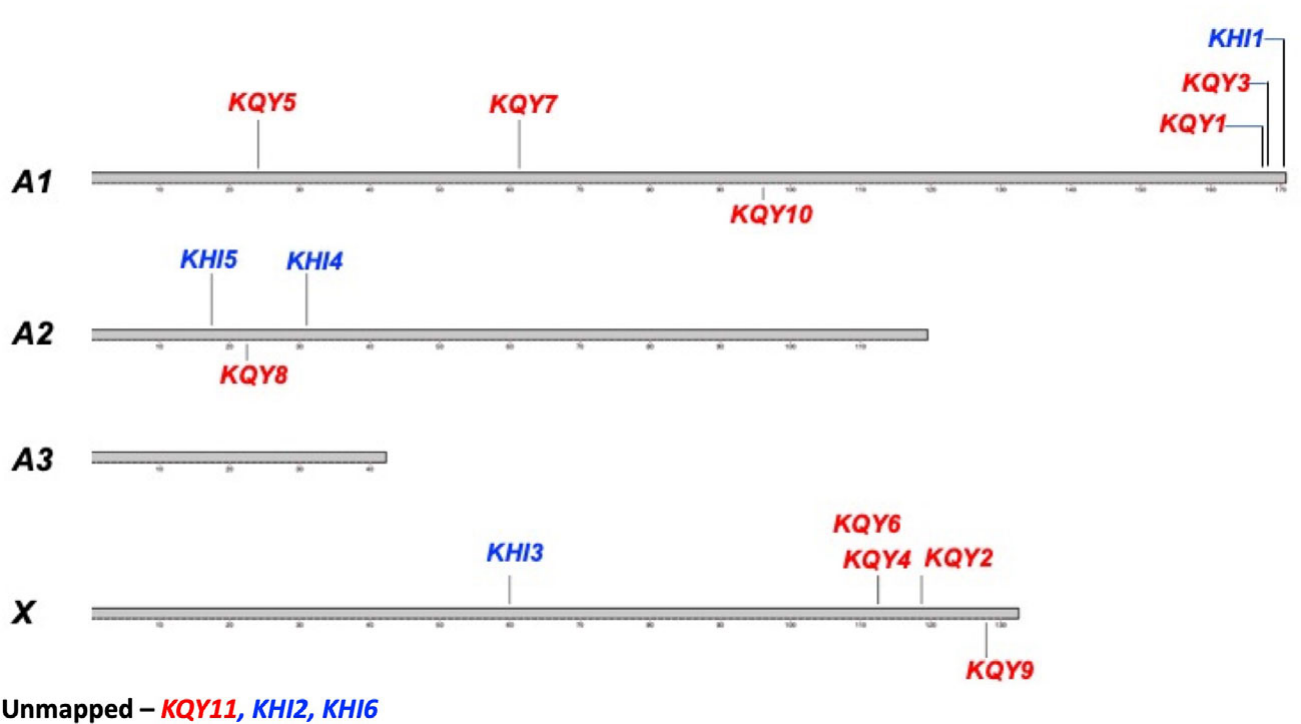
Placement of the *KQY* and *KHI* genes on the pea aphid chromosomes. The pea aphid chromosomes A1, A2, A3, and X. *KQY* genes are colored in red and the *KHI* genes are colored in blue. *KQY11, KHI2*, and *KHI6* are unmapped.

The pea aphid genome consists of four different chromosomes. The gene for KQY1a protein (gi|241896885) is anchored on the A1 chromosome in the *A. pisum* strain AL4f genome (GCF_005508785.1) (**Table 4**). Each of the pea aphid KQY genes have been found placed within the genome except for KQY11. KQY genes can be found placed on chromosome A1, A2, and X but not A3 (**Table 4**, **Figure 3**). Two of the KHI genes, KHI2 and KHI6, were unable to be placed within the pea aphid genome, and the remaining KHI genes were also found on chromosome A1, A2, and X, but not A3 (**Table 4**, **Figure 3**).

**Table 4:** Genome locations of KQY and KHI families.

Gene Family	Gene/Isoform Designation	Gene Locus	Chromosome	Location	Size (kb)exons (range bp)/introns (range bp)
**KQY**	KQY1a	LOC100158789	Chr A1 NC_042494.1	167,823,999–167,869,655	45.7 37(6-416)/36(180-5168)
	KQY1b	LOC100158789			
	KQY2a	LOC100302371	Chr X NC_042493.1	118,679,504–118,712,594	33.1
	KQY2b	LOC100302371			31.2 >13(5-420)/>12(555-9442)
	KQY3	LOC100301916	Chr A1 NC_042494.1	Complement 168,127,940–168,141,540	13,6 15(9-582)/14(281-5949)
	KQY4a	LOC100302370	Chr X NC_042493.1	Complement 112,529,404–112,604,186	74.8 32(5-389)/>31(203-10515)
	KQY4b	LOC100302370			
	KQY4c	LOC100302370			
	KQY4d	LOC100302370			
	KQY5a	LOC100302375			87.8 >27(6-399)/>26(377-43413)
	KQY5b	LOC100302376	Chr A1 NC_042494.1	24,100,856–24,188,627	87.8
	KQY6	LOC100302481 (NV12)	Chr X NC_042493.1	112,481,406–112,505,954	24.5
	KQY7	LOC100302485 (NV22)	Chr A1 NC_042494.1	Complement 61,298,211–61,309,480	11.3
	KQY8	LOC100302439	Chr A2 NC_042495.1	Complement 22,473,653–22,510,347 Length: 36,695 nt	36.7
	KQY9	LOC100302403	Chr X NC_042493.1	Complement 127,899,608–127,978,192	78.6
	KQY10	LOC100302381	Chr A1 NC_042494.1	96,124,545–96,133,787	9.2
	KQY11	LOC100302480	NW_021761267.1 unplaced		
**KHI**	KHI1a	LOC100159750	Chr A1 NC_042494.1	complement 170,686,182–170,699,132	13.0 12.8, 11(27-564)/10(170-2537)
	KHI1b	LOC100159750			
	KHI2a	LOC100302383	NW_021771857.1 unplaced		
	KHI2b	LOC100302383			
	KHI2c	LOC100302383			
	KHI3a	LOC100166702	Chr X NC_042493.1	59,926,126–59,940,736	14.6 13(6-355)/12(173-2296)
	KHI3b	LOC100166702			14.6 14(6-355)/13(95-2296)
	KHI4	LOC100570519 (ACYPI46154)	Chr A2 NC_042495.1	complement 31,024,034–31,037,869	13.9
	KHI5	LOC100534636	Chr A2 NC_042495.1	complement 17,449,710–17,453,573	18.9 >13(16-254)/>12(67-6096)
	KHI6	LOC100571631	NW_021770650.1 unplaced		

**Figure 4:**
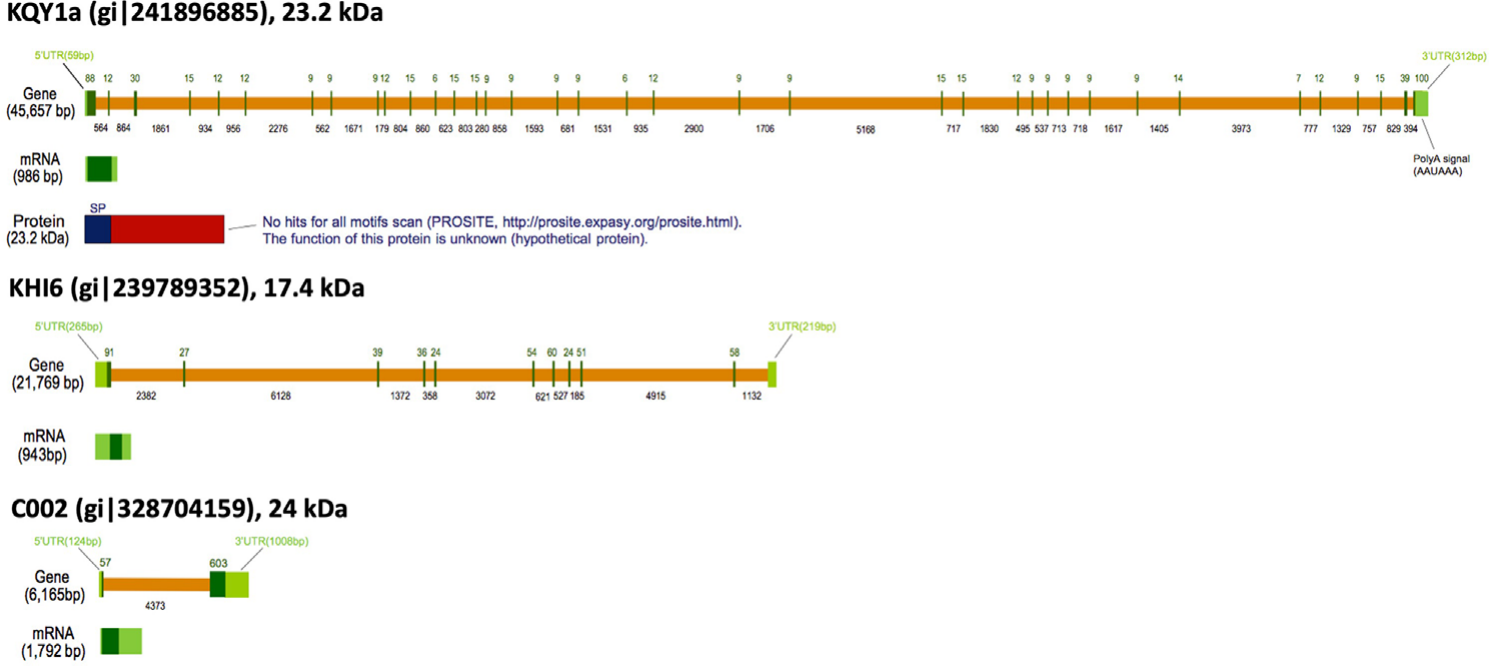
Gene structures of KQY1a and KHI6. C002 is shown for comparison. Diagrams are based on the graphic sequence display generated from the NCBI genome sequence viewer. Orange bars = introns; Green bars = exons.

*KQY1a* covers approximately 45.7 kbp, and the transcript is comprised of 37 relatively small exons (6−416 bp) (**Figure 4**). This structure of a large gene coding for a small protein using many miniature exons is also observed with other KQY gene family members (**Table 4**). KHI members are also generated from relatively large genes. The *KHI6* transcript (gi|239789352) is 943 bps long and comes from 10 exons in a gene that is 21.789 kbps long (**Figure 4**). The gene sizes of the mentioned gene families range from 12 kbp to 87 kbp. The first reported pea aphid effector/secretion protein, C002, shown here for contrast, has relatively small gene size (~6 kbp) with only two exons (**Figure 4**). No significant similar/conserved protein motifs and domains were found. The protein function of the gene family is unknown (hypothetical protein). A separate predicted locus (LOC100569066) can be found within an intron of *KQY2.* The gene product is highly conserved RAD50-interacting protein 1 (XP_016658051).

## Discussion

Here, we add to the characterization of candidate effectors of *A. pisum*, and, by sequence relatedness, possibly, *M. persicae* with the description of two families of genes, which by several criteria, appear to be variable secreted salivary gland proteins (Carolan et al., 2009; Carolan et al., 2011; Rao et al., 2013; Boulain et al., 2018). Twenty-seven protein candidates based on representative cDNAs could be assigned to either the KQY or KHI families, and most of the cDNA were represented in salivary gland RNAseq libraries. All of the loci, with exception of KQY2, were previously shown to have at least one isoform up-regulated in salivary gland in relation to alimentary tract expression, and all are predicted to encode secreted small molecular weight proteins (~12−28 kDa). Furthermore, peptides from a majority of the loci were detected in protein extractions of washed salivary glands, and one was detected in artificial feeding media. In a previous analysis, unidentified isoforms of three cassette effectors were detected in an artificial diet, including KQY2, which lacked clear evidence for salivary gland expression (Boulain et al., 2018).

The members of the two families were named cassette effectors due to the conserved N-terminal region, which harbors the signal secretion motif, and the divergent coding sequences distal to the secretory signal region. The model implies that novel coding sequences could be swapped on to the signal cassette, generating novel secreted proteins, which, in turn, can then facilitate the adaptation process of the aphid to new hosts or host varieties. The KQY genes can be grouped into three gene subfamilies that have, at least in part, expanded by gene duplication and divergence. KQY7 constitutes a single gene subfamily. Nonetheless, members of different families share sequence similarities beyond the coding regions indicating possible mosaic gene structure. The presence of a single KQY candidate from the related but distant green peach aphid (*M. persicae*) may be the result of amplification of a single gene during adaptation of pea aphids to various leguminous hosts. Whether cassette swapping was involved in adaption to a new host cannot be definitively stated. Analysis of various biotypes of *A. pisum* may reveal subspecies cassette gene content. Cassette effectors analogous to KQY and KHI have been previously identified in the Hessian fly genome, where the SSSGP-1 family share a similar structure (Chen et al., 2010), and domain swapping with secretory domains has been proposed, to name a few, to drive complexity in scorpion venom, in the evolution of plastid nuclear encoded proteins, and new virulence in nematodes (Tonkin et al., 2008; Vanholme et al., 2009; Wang et al., 2016). Exon shuffling has long been proposed, in itself, as one benefit of eukaryotic gene structure (Koonin et al., 2013; Smithers et al., 2019). The KQY and KHI genes are represented by varying numbers of mRNAs isoforms. However, definitive conclusions with regards to the levels of individual isoforms or loci remain unclear.

Some of the candidate cassette effector genes are quite large. *KQY1a*, as an example, is produced from a 986 base mRNA, which, in turn, is spliced from 46 kb of DNA, containing 37 exons and 36 introns. The gene sizes are not the largest, but, given the protein product, they are remarkable. The human gene for type III collagen, for example, is 44 kb and has 52 exons. However, the mRNA is 5460 bases, encoding a protein of 1446 amino acid residues in length, compared to the 986 mRNA and 204 aa products. *KQY4* and *KQY5* may be nearly twice the size of *KQY1a*. Further conclusions regarding KQY4 and KQY5 and some other gene of the candidate cassette effectors await improved genome sequencing and assembly. General conclusions regarding the arrangement of the genes may change due to future assembly improvements. The gene that can be mapped are scattered throughout the genome and, at present, only one pair are present in tandem (KQY4 and KQY6), despite the general view that highly evolving loci occur in multigenic loci. The contribution of cassette family genes to aphid adaptation awaits attempts to alter the expression of individual genes.

## Materials and Methods

### Pea Aphids, Salivary Glands, Proteins Collection

Pea aphid (*A. pisum*) clone LSR1 was maintained on *Vicia faba* at 20°C. Salivary glands of feeding adult aphid on the host plants were dissected following a protocol of the previous study (Mutti et al., 2006). For salivary gland protein extraction, the dissected salivary glands of *A. pisum* were stored in PBS solution with protease inhibitor cocktail (Roche) and centrifuged at 12,000 × g for 15 min at 4°C without tissue homogenization to avoid cellular proteins. After centrifugation and collecting supernatant, salivary gland proteins of the supernatant were precipitated with 20% TCA (v/v) and incubated at -20°C, overnight. The protein pellet was collected by centrifugation (1,500 × g for 10 min, 4°C) and then washed with 100% acetone 3 times and allowed the protein pellet to air dry. The protein pellet was dissolved in SDS-PAGE sample buffer [0.25 M Tris-HCl (pH6.8), 50% glycerol, 5% SDS, and 5% β-mercaptoethanol] for protein separation by 1-D SDS-PAGE for proteome analysis.

### Saliva Collection From Artificial Diet

Synthetic diet preparation and saliva collection were conducted under aseptic conditions (Will et al., 2007). Pea aphid saliva collection plates were prepared by stretching sterilized parafilm over the bottom of the 100 by 15 mm plastic petri dishes. Parafilm sheet surface sterilized and exposed to UV light for 30 min and the parafilms were stretched to 50% of the original size. Five milligrams chemically defined synthetic diet (35% sucrose solution) was placed on the stretched parafilm and cover with the other sterilized stretched parafilm (**Figure 1A**). Fifteen aphid saliva collection plates (approximately 1,600 pea aphid on each plate) were prepared for the secreted saliva collection from the synthetic diet. The diet from a 24 h collection period was pooled to give a volume approximately 75 ml, followed by concentration using a Vivaspin concentrator (GE Healthcare) with 3,000 molecular weight cut-off PES membrane at 4°C. The concentrated proteins were separated by 1-D SDS-PAGE and visualized with Coomassie blue R-250.

### In Gel Sample Preparation for Mass Spectrometry

For salivary gland proteome analysis, we have identified salivary gland proteins using by 1-D GeLC-MS/MS proteome approach. Proteins from salivary gland tissues and artificial diet were separated on 8%–16% Tris-HCl precast gel (Bio-Rad) in a Mini-Protean Electrophoresis Unit (Bio-Rad) and stained with Coomassie blue R-250 (**Figure 1A**). The stained protein bands of interest were excised using sterile surgical blades and the gel slices (no larger than 2 × 5 mm) were transferred to individual 1.5 ml microcentrifuge tubes with 10 μl HPLC grade water to prevent dehydration and prepared In-gel digestion. Proteins in the gel slices were reduced with 10 mM DTT in 200 mM ammonium bicarbonate at 60°C for 15 min, and then subjected to amidation in 20 mM iodoacetamide in 200 mM ammonium bicarbonate at room temperature in the dark for 30 min. The gel pieces were washed with 200 mM ammonium bicarbonate/50% acetonitrile (v/v) before addition of 250 ml of acetonitrile and incubation at room temperature for 15 min. The remaining solvent was removed, and the gel slices were completely dried using SpeedVac system (Thermo Fisher Scientific). The proteins in the gel slices were digested with 5 ng/ml sequencing grade modified porcine trypsin (Promega) in 200 mM ammonium bicarbonate/10% acetonitrile (v/v) at 55°C for 2 h. Trypsin was inactivated by adding 0.1% trifluoroacetic acid after protein digestion and the supernatant was transferred into 0.5 ml microcentrifuge tube for mass spectrometric analysis.

### Capillary Liquid Chromatography-Mass Spectrometry Analysis for Protein Identification

Samples were analyzed by LC-MS/MS using a NanoAcquity chromatographic system (Waters Corp., Milford, MA) coupled to an LTQ-FT mass spectrometer (ThermoFinnigan, Bremen, Germany). Peptides were separated on a reverse-phase C_18_ column, 5 cm, 500 µm I.D. (CVC Microtech). A gradient was developed from 1% to 40% B (99.9% acetonitrile, 0.1% formic acid) in 50 min, ramped to 95% B in 4 min and held at 95% B for 5 min at a flow rate of 20 µl/min with solvents, A (99.9% H2O, 0.1% formic acid) and B. NanoAquity UPLC Console (Waters Corp., Version 1.3) was used to execute the injections and gradients. The ESI source was operated with spray voltage of 2.8 kV, a tube lens offset of 160 V and a capillary temperature of 200°C. All other source parameters were optimized for maximum sensitivity of the YGGFL peptide MH^+^ ion at m/z 556.27. The instrument was calibrated using an automatic routine based on a standard calibration solution containing caffeine, peptide MRFA, and Ultramark 1621 (Sigma). Data-dependent acquisition method for the mass spectrometer (configured version LTQ-FT 2.2) was set up using Xcalibur software (ThermoElectron Corp., Version 2.0). Full MS survey scans were acquired at a resolution of 50,000 with an Automatic Gain Control (AGC) target of 5×10^5^. Five most abundant ions were fragmented in the linear ion trap by collision-induced dissociation with AGC target of 2×10^3^ or maximum ion time of 300 ms. The ion selection threshold was 500 counts. The LTQ-FT scan sequence was adapted from the reference (Olsen and Mann, 2004).

### Database Searches

MS/MS spectra were analyzed using Mascot (Matrix Science, London, UK; Version 2.3). Mascot was set up to search the SwissProt database and our pea aphid salivary gland transcriptome data of *A. pisum* assuming the trypsin digestion. Search was performed with a fragment ion mass tolerance of 0.20 Da and a parent ion tolerance of 20 PPM. Iodoacetamide derivative of cysteine was specified as a fixed modification. Oxidation of methionine was specified as a variable modification. Scaffold software (Version 3.6, Proteome Software Inc., Portland, OR) was used to validate MS/MS based peptide and protein identifications. Peptide identification from the MS/MS data was performed using the MASCOT to correlate the data against NCBI non-redundant database and our salivary gland transcriptome data of *A. pisum.* To improve peptide identification accuracy, the results of protein identification were validated by multiple search engines (Mascot, Sequest and X! Tandem) using Scaffold software. Peptide identifications were accepted if they could be established at greater than 50.0% probability as specified by the Peptide Prophet algorithm (Keller et al., 2002). Protein identifications were accepted if they could be established at greater than 99.0% probability and contained at least two identified peptides. Protein probabilities were assigned by the Protein Prophet algorithm (Nesvizhskii et al., 2003). Proteins that contained similar peptides and could not be differentiated based on MS/MS analysis alone were grouped to satisfy the principles of parsimony.

### Protein Sequence and Domain/Motif Analysis

The amino acid sequence of the proteins of the gene family was analyzed with ClustalW alignment program for the gene family protein grouping (https://www.genome.jp/tools-bin/clustalw) using the slow parameters of a 10.0 gap open penalty and a 0.1 gap extension penalty with the BLOSUM (for protein) weight matrix. The amino acid alignment was produced by T-Coffee using default parameters (ebi.ac.uk/Tools/msa/tcoffee/) and illustrated using BoxShade (embnet.vital-it.ch/software/BOX_form.html). The MS-identified protein sequences were analyzed with the ScanProsite and SMART program at the ExPaSy (http://expasy.org/), and EMBL (http://smart.embl-heidelberg.de/) for the domain/motif analysis to predict protein functions. Signal peptide of the all MS-identified proteins was predicted by using SignalP 4.1 server (http://www.cbs.dtu.dk/services/SignalP/) with a eukaryote D-cutoff value of 0.6. The pea aphid genome map was produced using karyoploteR (bioconducter.org/packages/release/bioc/html/karyoploteR.html) (Gel and Serra, 2017). Transcript similarity analysis was done using BLASTN comparing two or more sequences (https://blast.ncbi.nlm.nih.gov/Blast.cgi?PAGE_TYPE=BlastSearch). The KQY3 transcript without the secretory peptide and polyA regions was analyzed using BLASTN against single members of the KQY gene families also without their signal peptide and polyA nucleotides.

## Data Availability Statement

The datasets presented in this study can be found in online repositories. The names of the repository/repositories and accession number(s) can be found in **Supplemental Table 1**, further inquiries can be directed to the corresponding author/s.

## Author Contributions

MD and JO are co-first authors. All authors contributed to the article and approved the submitted version.

## Conflict of Interest

The authors declare that the research was conducted in the absence of any commercial or financial relationships that could be construed as a potential conflict of interest.

The reviewer SH declared a past co-authorship with one of the authors AS to the handling editor.

## Supplementary Material

Supplemental Figure 1 Full amino acid sequence alignment of the KQY salivary gland secretion protein candidates. The alignment was produced with the ClustalW multiple alignment program.

Supplemental Figure 2 Full amino acid sequence alignment of the KHI salivary gland secretion protein candidates. The alignment was produced with the ClustalW multiple alignment program.

Supplemental Figure 3 Pairwise BLASTP analysis of KQY family. Number indicates probability of match by chance (expect value). Cells of the same color indicate member of possible gene family at probability below 1e-05. Only one isoform was used for each gene.

Supplemental Figure 4 Alignment of KQY3 transcript with other members of the KQY family by BLAST. Colored boxes indicate alignment scores above 40. Red ticks indicate relative location of the stop codon for each gene.
