# Supplementary material for: Big Genes, Small Effectors: Pea Aphid Cassette Effector Families Composed From Miniature Exons
Source: Front Plant Sci. 2020 Sep 2;11:1230. doi: 10.3389/fpls.2020.01230 (PMC7495047; doi:10.3389/fpls.2020.01230)
Supplement: Supplementary file 1 [file Table_1.docx]

| **Supplemental Table 1**. Secretion protein candidates (10−60KDa) from the *A. pisum* salivary gland proteome analysis using 1-D geLC-MS/MS | | | | | | |
| --- | --- | --- | --- | --- | --- | --- |
| **Protein Name** | **NCBI Accession Number** |  | **# of Peptides (Unique)** | **% Coverage** | **Signal Peptide Probability**  **(D-score)** | **Mw (KDa)** |
| KQY9  LOC100302403 | NP_001156509 |  | 3(3) | 32 | 0.903 | 13.3 |
| KQY9  LOC100302403 | NP_001156509 |  | 3(3) | 24 | 0.903 | 13.3 |
| Hypothetical protein ACYPI003695  LOC100162547 | NP_001280471 |  | 2(2) | 23 | 0.79 | 13.9 |
| ACYPI003695 | NP_001280471 |  | 2(2) | 23 | 0.797 | 13.9 |
| Chemosensory protein-like precursor | NP_001119649 |  | 1(1) | 8 | 0.794 | 15.3 |
| KQY3  LOC100301916 | NP_001153885 |  | 2(1) | 13 | 0.729 | 16.1 |
| Hypothetical protein LOC100165393 | gi\|240849625 |  | 6(5) | 49 | 0.805 | 16.1 |
| Chemosensory protein-like precursor | gi\|187125206 |  | 3(3) | 23 | 0.631 | 16.4 |
| Hypothetical protein LOC100302485 | gi\|254281288 |  | 2(2) | 14 | 0.664 | 17.3 |
| KHI6  LOC100571631 | NP_001233103 |  | 4(4) | 44 | 0.778 | 17.4 |
| KQY11  LOC100302480 | NP_001156816 |  | 3(3) | 24 | 0.847 | 17.4 |
| KQY2a  LOC100302371 | BAH72568 |  | 9(8) | 47 | 0.685 | 17.8 |
| Hypothetical protein LOC100163734 | NP_001155588 |  | 3(3) | 31 | 0.694 | 17.9 |
| KQY2b  LOC100302371 | NP_001156348 |  | 8(6) | 36 | 0.685 | 17.9 |
| Hypothetical protein | gi\|193659833 |  | 3(3) | 25 | 0.58 | 18.7 |
| ACYPI001541 | gi\|239788714 |  | 3(3) | 25 | 0.559 | 18.9 |
| ACYPI000294 | gi\|239799135 |  | 9(8) | 54 | 0.798 | 19.3 |
| Hypothetical protein LOC100158873 | gi\|241896896 |  | 9(8) | 54 | 0.798 | 19.3 |
| KHI3a  LOC100166702 | NP_001156548 |  | 12(9) | 56 | 0.744 | 19.8 |
| Putative defense protein Hdd1-like | gi\|193587386 |  | 2(2) | 10 | 0.805 | 19.8 |
| ACYPI002876 | gi\|239787958 |  | 2(2) | 10 | 0.805 | 19.8 |
| KHI1a  ACYPI001099  LOC100159750 | NP_001155863 |  | 4(3) | 33 | 0.753 | 20.3 |
| Armet-like protein | gi\|193714968 |  | 6(6) | 36 | 0.816 | 20.4 |
| Hypothetical protein LOC100164214 | gi\|240848531 |  | 12(10) | 44 | 0.703 | 20.9 |
| KQY5b  LOC100302376 | NP_001156435 |  | 8(8) | 45 | 0.842 | 21.8 |
| KHI5  LOC100534636 | NP_001191953 |  | 6(6) | 38 | 0.805 | 21.9 |
| Hypothetical protein LOC100159619 | gi\|269784619 |  | 3(3) | 22 | 0.863 | 22.7 |
| Hypothetical protein | gi\|193702374 |  | 7(5) | 49 | 0.771 | 22.7 |
| ACYPI38240 | gi\|239788286 |  | 2(2) | 12 | 0.882 | 22.9 |
| KQY1a  ACYPI000223  LOC100158789 | NP_001155914 |  | 17(14) | 66 | 0.798 | 23.2 |
| KHI2a  LOC100302383 | BAH71618 |  | 2(2) | 14 | 0.798 | 23.3 |
| hypothetical protein LOC100159932 | gi\|242247644 |  | 6(6) | 38 | 0.865 | 23.6 |
| ACYPI007406 | gi\|239793559 |  | 7(5) | 47 | 0.771 | 23.6 |
| Hypothetical protein LOC100167237 | gi\|240849045 |  | 2(2) | 14 | 0.697 | 23.7 |
| KHI2b  LOC100302383 | NP_001156448 |  | 2(2) | 14 | 0.798 | 23.8 |
| Hypothetical protein | gi\|239790064 |  | 2(2) | 14 | 0.798 | 23.8 |
| ACYPI008569 | gi\|239789136 |  | 9(7) | 40 | 0.85 | 24 |
| C002 | XM_001948323 |  | 5(4) | 33 | 0.612 | 24 |
| Hypothetical protein LOC100168831 | gi\|240848667 |  | 3(3) | 18 | 0.57 | 24.1 |
| ACYPI009500 | gi\|239790223 |  | 3(3) | 18 | 0.57 | 24.1 |
| Hypothetical protein LOC100168726 | gi\|240848575 |  | 2(2) | 20 | 0.769 | 24.4 |
| ACYPI001606 | gi\|239790177 |  | 3(3) | 18 | 0.661 | 25.5 |
| Hypothetical protein LOC100160301 | gi\|240848781 |  | 3(3) | 18 | 0.661 | 25.5 |
| Hypothetical protein LOC100302481 | gi\|254039685 |  | 10(9) | 34 | 0.82 | 26.4 |
| Hypothetical protein | gi\|239788195 |  | 10(9) | 33 | 0.82 | 26.4 |
| KQY10  LOC100302381 | NP_001156441 |  | 1(1) | 6 | 0.377 | 26.7 |
| KQY4c  LOC100302370 | BAH72627 |  | 10(9) | 45 | 0.854 | 27.5 |
| Hypothetical protein | gi\|193700110 |  | 7(6) | 36 | 0.623 | 28.1 |
| KQY4a  LOC100302370 | NP_001156343 |  | 10(9) | 43 | 0.854 | 28.3 |
| Endoplasmic reticulum resident protein 29-like | gi\|193678927 |  | 7(7) | 28 | 0.734 | 28.5 |
| ACYPI000995 | gi\|239789964 |  | 7(7) | 28 | 0.734 | 28.5 |
| Translocon-associated protein subunit alpha-like | gi\|193669405 |  | 2(2) | 10 | 0.89 | 30 |
| ACYPI006658 | gi\|239788342 |  | 2(2) | 10 | 0.89 | 30.1 |
| Hypothetical protein | gi\|193671558 |  | 4(3) | 18 | 0.791 | 30.3 |
| Similar to CG8132 CG8132-PA | gi\|193695366 |  | 1(1) | 15 | 0.598 | 31.2 |
| Gamma interferon inducible lysosomal thiol reductase (GILT)-like isoform 1 | gi\|193647949 |  | 2(2) | 9 | 0.902 | 32.7 |
| Hypothetical protein LOC100159063 | gi\|240849255 |  | 4(4) | 19 | 0.783 | 34.8 |
| Cathepsin B-like cysteine proteinase 5-like | gi\|193606095 |  | 4(4) | 13 | 0.592 | 37.7 |
| ACYPI001175 | gi\|239790303 |  | 4(4) | 13 | 0.592 | 37.8 |
| Cathepsin B-1674 precursor | gi\|209863086 |  | 3(3) | 13 | 0.56 | 38.2 |
| ACYPI000012 | gi\|239799412 |  | 3(3) | 7 | 0.56 | 38.2 |
| anterior fat body-like protein | gi\|193669147 |  | 3(3) | 15 | 0.647 | 39.2 |
| DnaJ homolog subfamily B member 11-like | gi\|193617724 |  | 2(2) | 11 | 0.627 | 40.8 |
| Disulfide-isomerase (PDI) A6 | gi\|193695172 |  | 12(10) | 41 | 0.761 | 42.6 |
| Cathepsin D | gi\|237874218 |  | 3(3) | 11 | 0.736 | 42.8 |
| Proclotting enzyme-like isoform 1 | gi\|193575579 |  | 4(4) | 15 | 0.55 | 43.1 |
| Gelsolin-like isoform 1 | gi\|193716193 |  | 8(8) | 27 | 0.83 | 44.4 |
| Hypothetical protein | gi\|193671765 |  | 7(7) | 26 | 0.78 | 45.3 |
| Similar to calreticulin | gi\|193575611 |  | 20(18) | 48 | 0.52 | 46.4 |
| Endoplasmic reticulum protein 44-like | gi\|193709296 |  | 8(8) | 28 | 0.609 | 47.1 |
| Alpha-N-acetylgalactosaminidase-like | gi\|193664693 |  | 4(3) | 11 | 0.785 | 48.4 |
| Imaginal disk growth factor (IDGF) precursor | gi\|274327724 |  | 5(5) | 16 | 0.805 | 48.7 |
| Protein disulfide-isomerase (PDI) A3-like | gi\|193713655 |  | 25(22) | 60 | 0.885 | 55.3 |
| DnaJ homolog subfamily C member 3-like | gi\|193620476 |  | 7(7) | 20 | 0.721 | 56.7 |
| Protein disulfide-isomerase (PDI)-like | gi\|193656973 |  | 47(34) | 74 | 0.84 | 57.3 |
| Protein ERGIC-53-like isoform 1 | gi\|193652640 |  | 3(3) | 10 | 0.726 | 57.8 |
