# Supplementary figures and images for: Big Genes, Small Effectors: Pea Aphid Cassette Effector Families Composed From Miniature Exons
Source: Front Plant Sci. 2020 Sep 2;11:1230. doi: 10.3389/fpls.2020.01230 (PMC7495047; doi:10.3389/fpls.2020.01230)

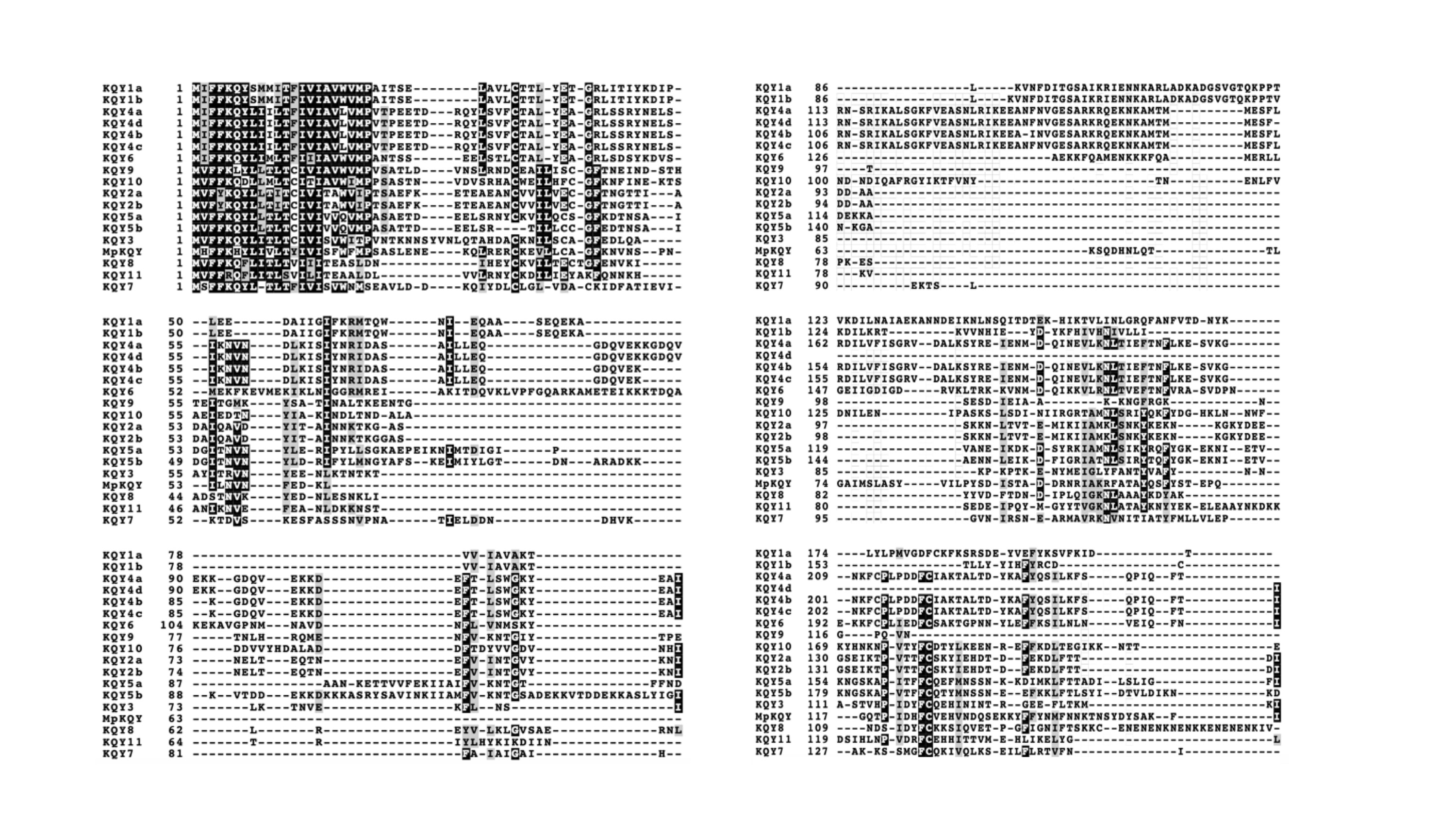

Supplement: Supplemental Figure 1 — Full amino acid sequence alignment of the KQY salivary gland secretion protein candidates. The alignment was produced with the ClustalW multiple alignment program. [file Image_1.jpeg]

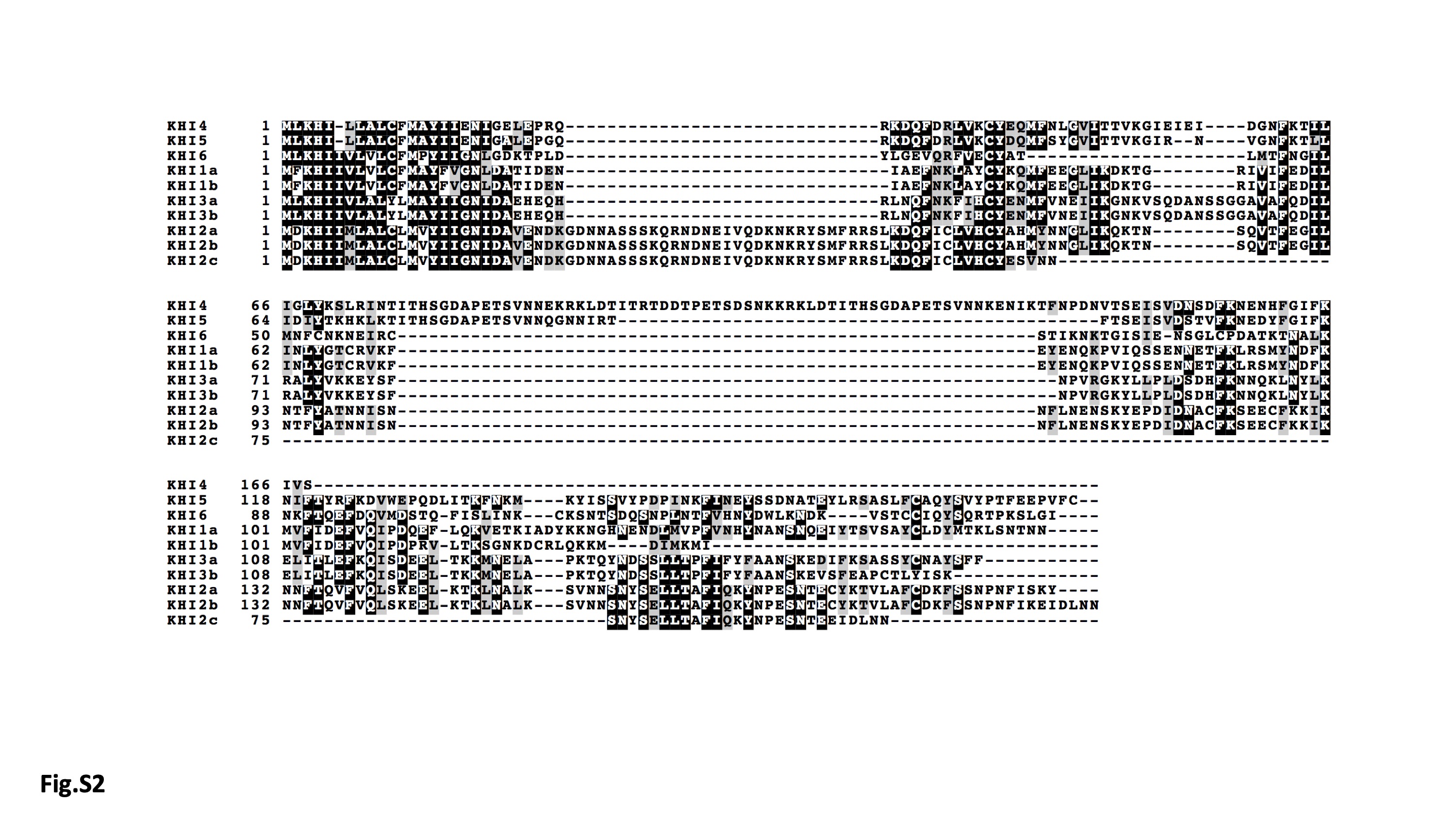

Supplement: Supplemental Figure 2 — Full amino acid sequence alignment of the KHI salivary gland secretion protein candidates. The alignment was produced with the ClustalW multiple alignment program. [file Image_2.jpeg]

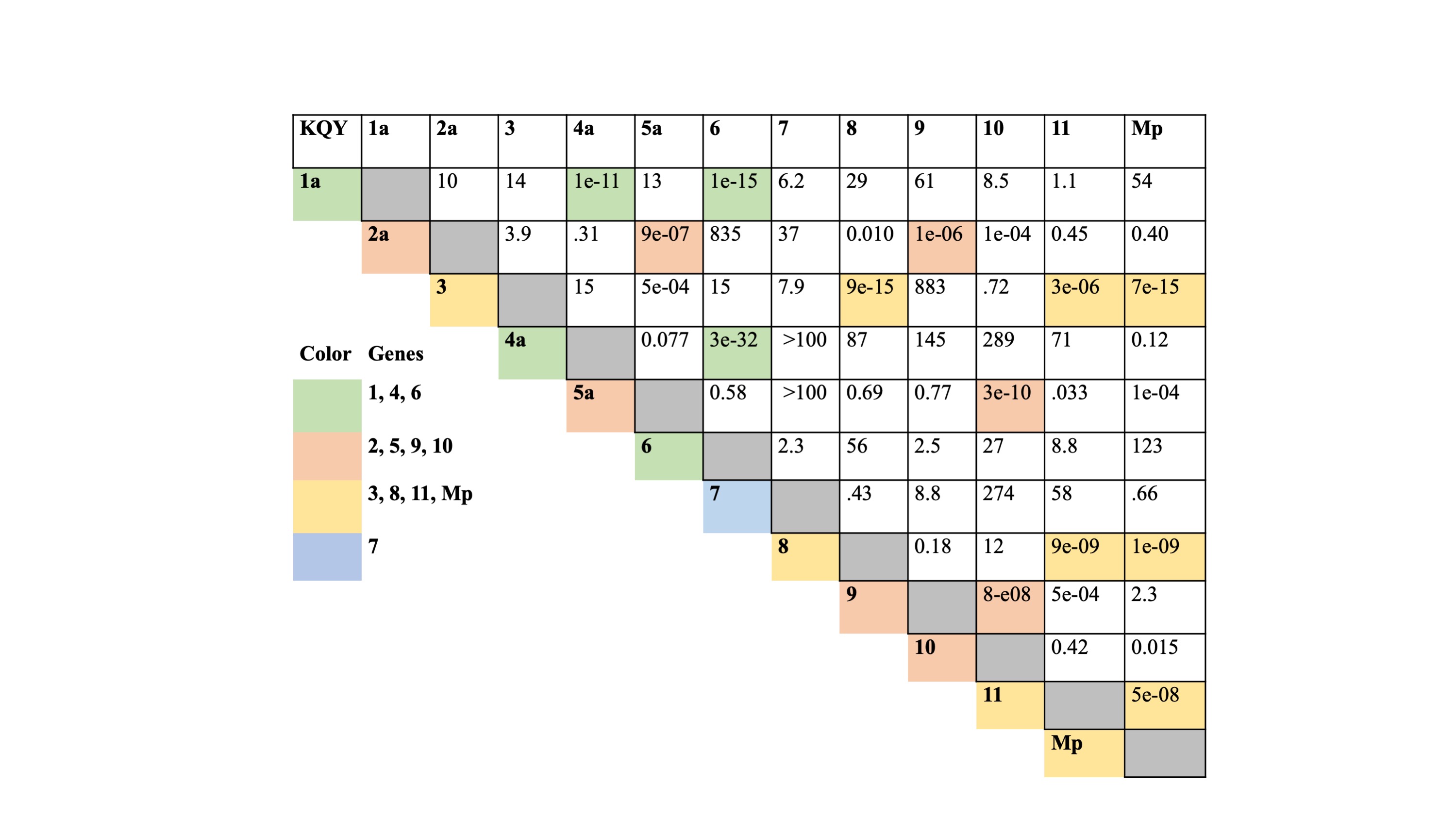

Supplement: Supplemental Figure 3 — Pairwise BLASTP analysis of KQY family. Number indicates probability of match by chance (expect value). Cells of the same color indicate member of possible gene family at probability below 1e-05. Only one isoform was used for each gene. [file Image_3.jpeg]

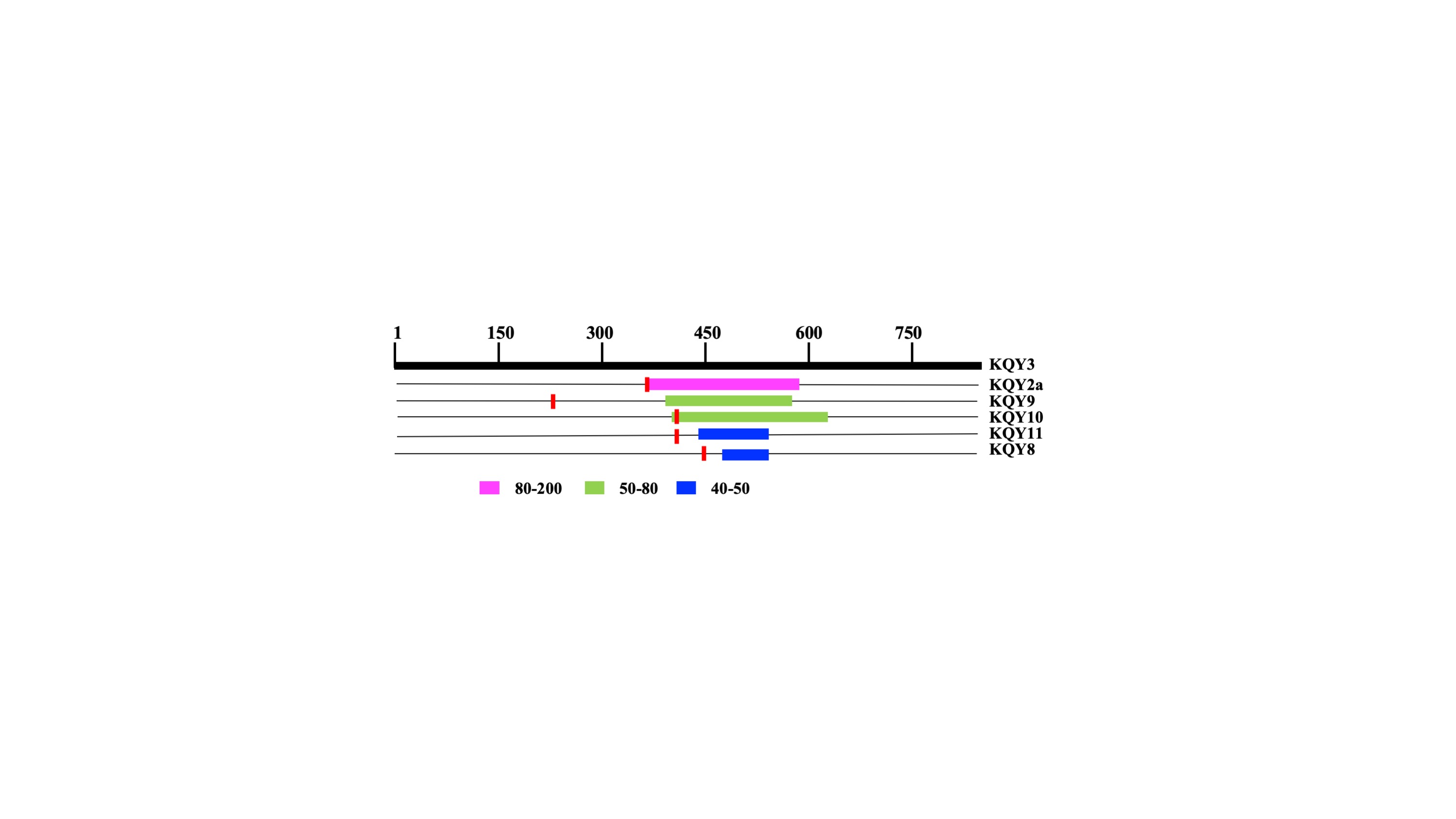

Supplement: Supplemental Figure 4 — Alignment of KQY3 transcript with other members of the KQY family by BLAST. Colored boxes indicate alignment scores above 40. Red ticks indicate relative location of the stop codon for each gene. [file Image_4.jpeg]
